# Well-protected quantum state transfer in a dissipative spin chain

**DOI:** 10.1038/s41598-018-26220-y

**Published:** 2018-05-21

**Authors:** Naghi Behzadi, Abbas Ektesabi, Bahram Ahansaz

**Affiliations:** 1 Research Institute for Fundamental Sciences, University of Tabriz, Tabriz, Iran; 2 Physics Department, Azarbaijan Shahid Madani University, Tabriz, Iran

## Abstract

In this work, a mechanism is investigated for improving the quantum state transfer efficiency in a spin chain, which is in contact with a dissipative structured reservoir. The efficiency of the method is based on the addition of similar non-interacting auxiliary chains into the reservoir. In this way, we obtain the exact solution for the master equation of the spin chain in the presence of dissipation. It is found out that entering more auxiliary chains into the reservoir causes, in general, the better improvement of the fidelity of state transfer along the mentioned chain. Furthermore, it is reveal that the protocol has better efficiency for a chain with longer length. Therefore, by this method, quantum state transfer along a linear chain with an arbitrary number of qubits, can be well-protected against the dissipative noises.

## Introduction

The high-fidelity transmission of quantum states from one location to another in a quantum network through a quantum channel is an important task in quantum information processing. This is so because any performance of a quantum information processing task inside a quantum computer needs to exchange quantum information between distant nodes. Among the various physical systems, quantum spin chains are the best-known ones that can serve as quantum channels. After the pioneer work of Bose^1^, in which an unmodulated ferromagnetic spin chain with nearest neighbor Heisenberg interaction was proposed as a channel for short range quantum communication, various theoretical frameworks were proposed to increase the transmission fidelity in quantum state transfer (QST)^2–4^ and even to achieve perfect state transfer (PST) in spin chains^5–10^. Since PST in spin chains with uniform nearest neighbor couplings is possible only for the chains with two and three spins, therefore, PST in longer chains is achievable by properly engineering and modulating of these couplings^5,6,9^. On the other hand, the necessity of engineering the coupling strengths in order to achieve PST in spin networks, which in turns leads to the increment of the complexity of the system, can be removed by taking phase modulated uniform couplings^11,12^. Also, exploiting partial collapsing measurements can improve the QST in spin chains with uniform nearest neighbor couplings^13,14^. In addition, PST has been recently investigated using discrete-time quantum walk approach in refs^15,16^.

On the other hand, since any real system is inevitably subjected to its surrounding environment, achieving QST with high fidelity in the presence of noise and dissipation effects is an outstanding challenge in quantum channels. So it would be important to consider possible methods to minimize or eliminate these unwanted effects on the QST efficiency, as considered recently in^4,17–30^.

In this paper, we propose a theoretical approach to achieve high fidelity transmission of a quantum state in a linear spin chain which is in contact with a dissipative structured reservoir. It is assumed that the PST is achievable for the isolated spin chain due to the same pre-engineered nearest-neighbor couplings discussed in the refs^9,31^. The performance of the method is based on the enterance of other similar auxiliary spin chains, without direct interaction with each other, into the reservoir. In this direction, we provide the analytical solution for the dynamics of the chains immersed in the reservoir. It is found out that increasing the number of auxiliary chains leads to access to a high fidelity state transfer. Furthermore, it is figured out that for a chain with more qubits we have a better decoupling of the unitary dynamics of the chain from the dissipation, which means that the protocol has better efficiency for the chains with longer length.

In the following sections, we first review the PST in a spin chain according to the refs^9,31^. In the next step, the exact dynamics of the system in the presence of dissipative noises is obtained and consequently, the mechanism for protection of QST process against the dissipative noises, in the spin chain, is investigated. Finally, the paper is ended by a brief conclusion.

## Results

### Protection process in the presence of dissipation

**Figure 1:**
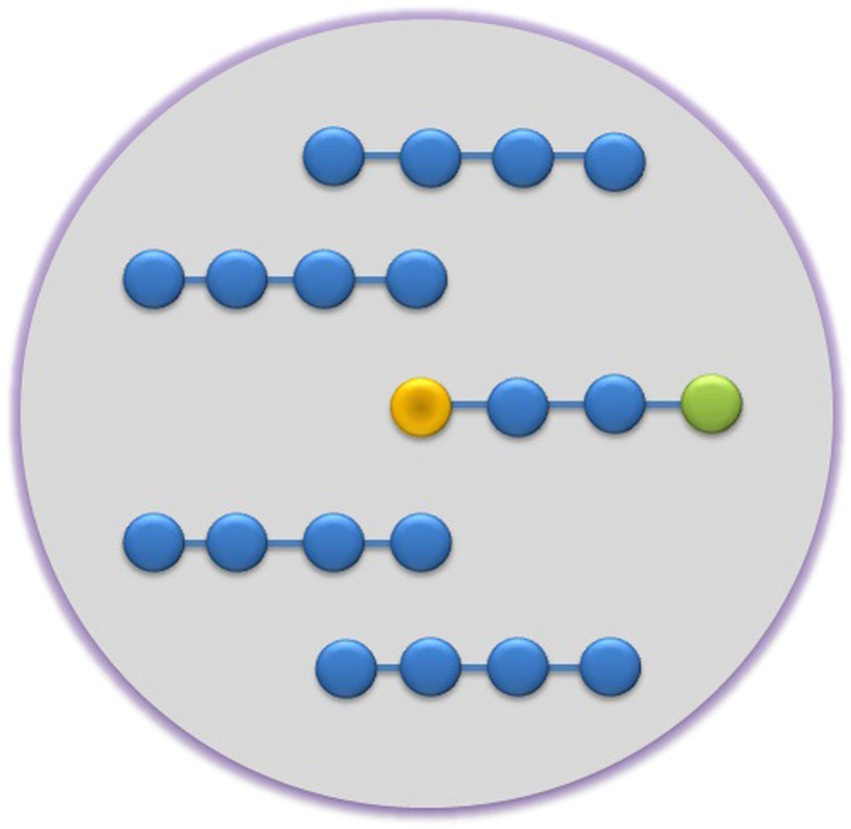
A schematic representation of a spin chain in the presence of, for example, four similar auxiliary chains contained in the reservoir.

In this stage we consider the spin chain as an open quantum system in which, the efficiency of state transfer process is degraded due to the existence of interaction between the chain and a dissipative structured reservoir. In other words, all of the qubits in the chain are contained in a common reservoir. We introduce the protection process by considering other *N* − 1 auxiliary similar chains with *M* spin, such that each of these chains is also involved in the above mentioned reservoir (see Fig. 1). It is assumed that there is no direct interaction between the chains. The Hamiltonian of the whole system reads as

1 $$\begin{array}{rcl}\hat{\bar{H}} & = & {\omega }_{0}\sum _{i=1}^{N}\sum _{j=0}^{M-1}{\sigma }_{i,j}^{+}{\sigma }_{i,j}^{-}\\  &  & +\sum _{i=1}^{N}\sum _{j=0}^{M-1}\sqrt{(j+1)(M-j-1)}({\sigma }_{i,j}^{+}{\sigma }_{i,j+1}^{-}+{\sigma }_{i,j}^{-}{\sigma }_{i,j+1}^{+})\\  &  & +\sum _{k}{\omega }_{k}{b}_{k}^{\dagger }{b}_{k}+\sum _{i=1}^{N}\sum _{j=0}^{M-1}\sum _{k}({g}_{k,j}{\sigma }_{i,j}^{+}{b}_{k}+{g}_{k,j}^{\ast }{\sigma }_{i,j}^{-}{b}_{k}^{\dagger }),\end{array}$$

where *ω*_0_ is the transition frequency, *b*_*k*_ (${b}_{k}^{\dagger }$) is the annihilation (creation) operator for the *k*th field mode with frequency *ω*_*k*_. In the above equation, we have introduced the site-dependent coupling strength *g*_*k*,*j*_ as the coupling constant between the *k*th field mode and the qubit located at site *j* of the each chain, defined as

2 $${g}_{k,j}={g}_{k}{\tilde{K}}_{0}(j),$$

where ${\tilde{K}}_{0}(j)$ is defined as a Krawtchouk function for *l* = 0, in Eq. (38) (see Methods). As a further illustration, it is clear from end part of the Eq. (1) that the environment has identical interactions with all of the chains. It can depend on the configuration of the chains inside the environment. In fact, it can be found a configuration for the chains in such a way that the coupling of the environment to the chains are occurred homogeneously in a similar way. Roughly speaking, the environment could be considered as electromagnetic radiations inside an imperfect cavity formed by two identical spherical mirrors^32^. Obviously, the cavity modes have a Lorentzian spectral density^33^. Since the cavity has cylindrical symmetry so all of the chains in the cavity which are parallel to the cavity axis with equal radius distance and also with equal distances from the mirrors, are coupled to the cavity modes in the same way. Therefore, by this reason, a common Lorentzian environment which interacts with a spin chain^34,35^, can be coupled to the arbitrary number of the other similar chains in the same way. Taking the site-dependent coupling strength *g*_*k*,*j*_ as Eq. (2), leads to the exact solution of the master equation for the dynamics of the system. It should be noted that taking each of the ${\tilde{K}}_{l}(j)$ for *l* = 0, 1, 2, ..., *M* − 1, also gives the exact solution of the master equation. For the lowering operator we have ${\sigma }_{ij}^{-}={({\sigma }_{ij}^{+})}^{\dagger }\equiv |\bar{{\bf{0}}}\rangle \langle i,j|$ where $|\bar{{\bf{0}}}\rangle \equiv |\mathop{\underbrace{\mathrm{000...0...0}}}\limits_{N\times M}\rangle$ and $|i,j\rangle \equiv |\mathop{\underbrace{{\mathrm{000...1}}_{i,j}\mathrm{...0}}}\limits_{N\times M}\rangle$. In fact, |*i*, *j*〉 indicates that there exists an excitation in the *j*th site of the *i*th chain with *j*  = 0, 1, ..., *M* − 1 and *i* = 1, 2, ..., *N*. The states {|*i*, *j*〉} can be considered as a set of basis for the single excitation subspace of *N* similar chains, each of which having *M* identical qubits. By considering the unitary transformation $\hat{\bar{U}}={I}_{N\times N}\otimes \hat{U}$ ($\hat{U}$ is defined in Eq. (37) as a *M* × *M* -matrix with elements ${U}_{j,l}={\tilde{K}}_{l}(j)$) (see Methods), we can transform the Hamiltonian (1) to the diagonal form $\hat{\bar{H}}=\hat{\bar{U}}\hat{\bar{D}}{\hat{\bar{U}}}^{T}$, where

3 $$\hat{\bar{D}}={\rm{diag}}({E}_{j}^{i})\,\,{\rm{with}}\,\,{E}_{j}^{i}=(M-1)-2j,$$

with *j* = 0, 1, ..., *M* − 1 and *i* = 1, 2, ..., *N*. The columns of the matrix $\hat{\bar{U}}$ are the eigenvectors of $\hat{\bar{H}}$ and related to the Krawtchouk polynomials as follows

4 $$|{{\rm{\Phi }}}_{l}^{i}\rangle =\sum _{j=0}^{M-1}{U}_{j,l}|i,j\rangle =\sum _{j=0}^{M-1}{\tilde{K}}_{l}(j)|i,j\rangle .$$

From the orthogonality of $\hat{\bar{U}}$, the inverse relation follows as

5 $$|i,j\rangle =\sum _{l=0}^{M-1}{\tilde{K}}_{l}(j)|{{\rm{\Phi }}}_{l}^{i}\rangle .$$

So the Hamiltonian (1) in the basis $\{|{{\rm{\Phi }}}_{l}^{i}\rangle \}$ takes the following form

6 $$\hat{H}=\sum _{i=1}^{N}\sum _{l=0}^{M-1}({\omega }_{0}+{E}_{l}^{i})|{{\rm{\Phi }}}_{l}^{i}\rangle \langle {{\rm{\Phi }}}_{l}^{i}|+\sum _{k}{\omega }_{k}{b}_{k}^{\dagger }{b}_{k}+\sum _{i=1}^{N}\sum _{k}({g}_{k}{{\rm{\Xi }}}^{i+}{b}_{k}+{g}_{k}^{\ast }{{\rm{\Xi }}}^{i-}{b}_{k}^{\dagger }),$$

where

7 $${{\rm{\Xi }}}^{i+}={({{\rm{\Xi }}}^{i-})}^{\dagger }\equiv \sum _{j=0}^{M-1}{\tilde{K}}_{0}(j)|i,j\rangle \langle \bar{{\bf{0}}}|=|{{\rm{\Phi }}}_{0}^{i}\rangle \langle \bar{{\bf{0}}}\mathrm{|.}$$

**Figure 2:**
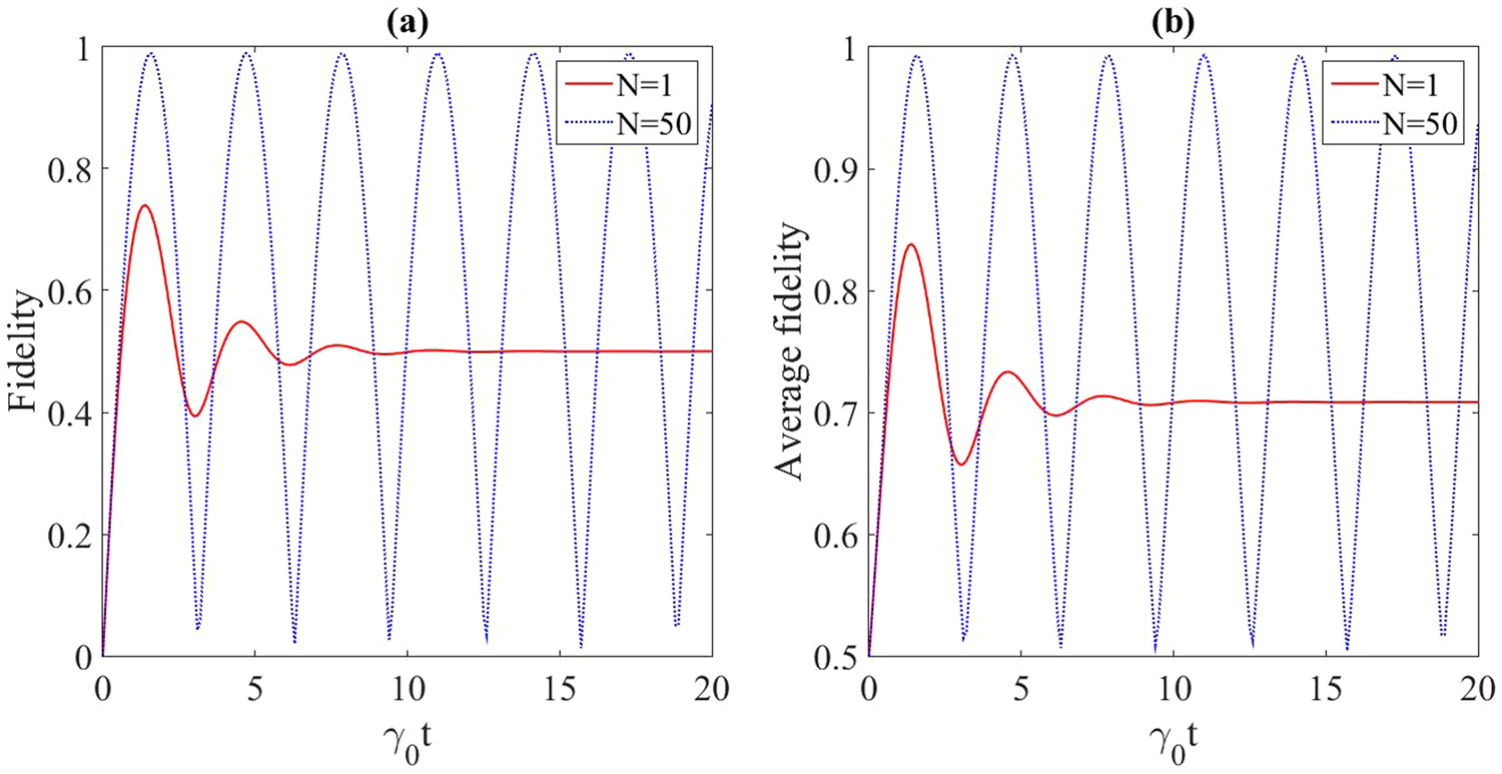
(**a**) Fidelity and (**b**) maximum average fidelity of the state transfer for the protected (*N* = 50) and unprotected (*N* = 1) two-qubit spin chain contained in the Lorentzian reservoir. The related parameters are *λ* = 50 (in units of *γ*_0_) and *ω*_0_ = 1 (in units of *γ*_0_). As illustrated in text, *N* = 50 means that there are 49 similar auxiliary chains in the reservoir for the aim of protection process.

**Figure 3:**
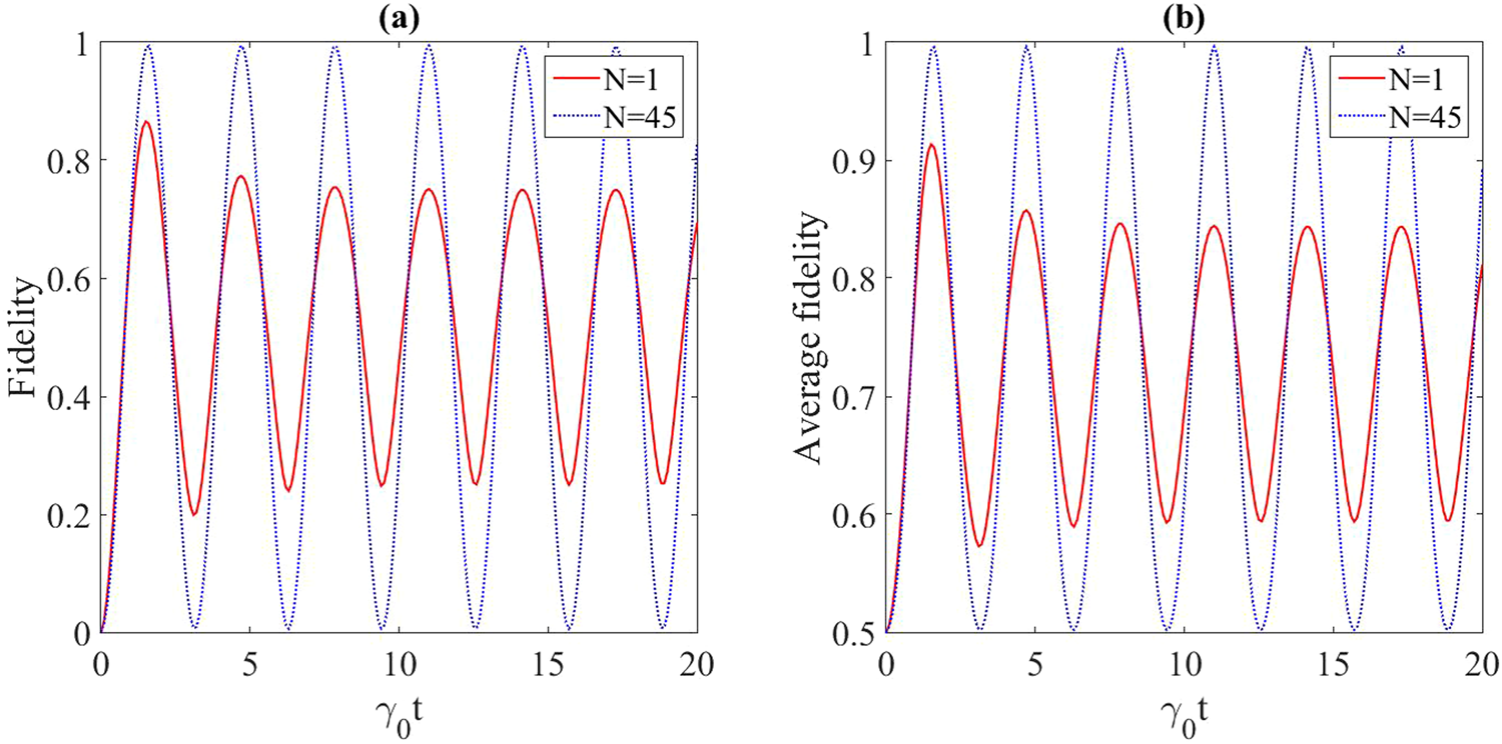
(**a**) Fidelity and (**b**) maximum average fidelity the of state transfer for the protected (*N* = 45) and unprotected (*N* = 1) three-qubit spin chain contained in the Lorentzian reservoir. The related parameters are *λ* = 50 (in units of *γ*_0_) and *ω*_0_ = 1 (in units of *γ*_0_).

**Figure 4:**
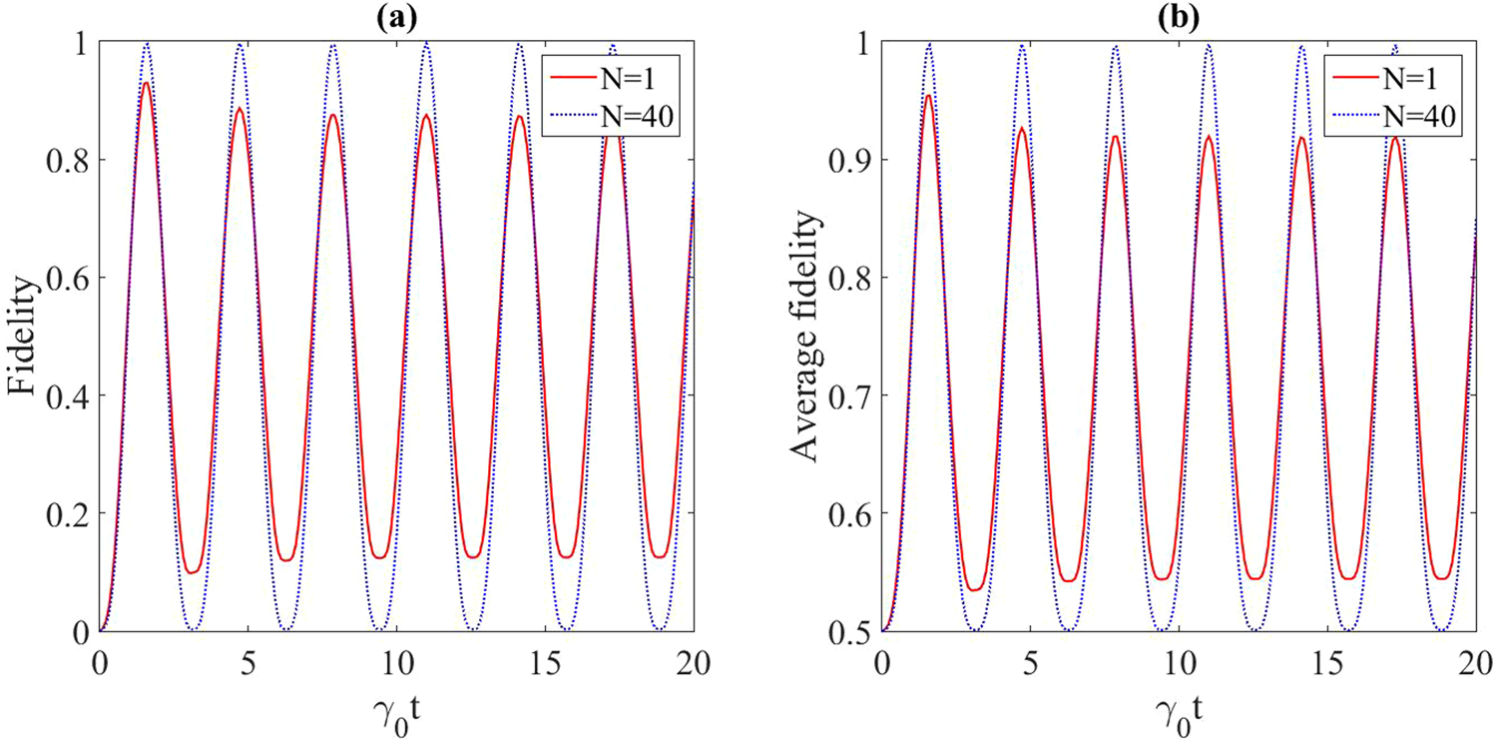
(**a**) Fidelity and (**b**) maximum average fidelity of the state transfer for the protected (*N* = 40) and unprotected (*N* = 1) four-qubit spin chain contained in the Lorentzian reservoir. The related parameters are *λ* = 50 (in units of *γ*_0_) and *ω*_0_ = 1 (in units of *γ*_0_).

It is clear from the Eq. (7) that the interaction of the system with the reservoir takes place collectively only through the eigenstate $|{{\rm{\Phi }}}_{0}^{i}\rangle$ and therefore, the *N*(*M* − 1) eigenstates of the system are decoupled from the reservoir. On the other hand, if we choose each of the ${\tilde{K}}_{l}(j)$ for *l* = 1, 2, 3, ..., *M* − 1 in Eq. (2), then the coupling of the system to the reservoir is provided only through the related $|{{\rm{\Phi }}}_{0}^{i}\rangle$ and therefore, the other *N*(*M* − 1) eigenstates are decoupled from the reservoir too. Then we conclude that the ratio of the dimensions of the coupled subspace to the reservoir and the decoupled one is $\frac{1}{M-1}$. This evidently demonstrates that for larger values of *M*, we have a better decoupling in this way (for example, see Figs 2–4).

Now, we consider the dynamics of the system by noting to the point that at initial time *t* = 0, there exists only a single excitation in one of the chains and the other *N* − 1 chains along with the reservoir are in their respective ground states. Let us assume that the initial state can be written, in general, as follows

8 $$|\psi \mathrm{(0)}\rangle =C\mathrm{(0)|}\bar{{\bf{0}}}{\rangle }_{S}\mathrm{|0}{\rangle }_{E}+\sum _{i=1}^{N}\sum _{l=0}^{M-1}{C}_{l}^{i}\mathrm{(0)|}{{\rm{\Phi }}}_{l}^{i}{\rangle }_{S}\mathrm{|0}{\rangle }_{E}\mathrm{.}$$

Since the Hamiltonian conserves the number of excitations in the system, the time-evolved state |*ψ*(*t*)〉 is

9 $$|\psi (t)\rangle =C\mathrm{(0)|}\bar{{\bf{0}}}{\rangle }_{S}\mathrm{|0}{\rangle }_{E}+\sum _{i=1}^{N}\sum _{l=0}^{M-1}{C}_{l}^{i}(t)|{{\rm{\Phi }}}_{l}^{i}{\rangle }_{S}\mathrm{|0}{\rangle }_{E}+\sum _{k}{C}_{k}(t)|\bar{{\bf{0}}}{\rangle }_{S}{\mathrm{|1}}_{k}{\rangle }_{E},$$

where |1_*k*_〉_*E*_ denotes the state of the reservoir with only one excitation in the *k*th mode. The time-dependent coefficients ${C}_{l}^{i}(t)$ and *C*_*k*_(*t*) are determined from the schrödinger equation $i\frac{d}{dt}|\psi (t)\rangle =\hat{\bar{H}}|\psi (t)\rangle$, as follows

10 $$\frac{d{C}_{0}^{i}(t)}{dt}=-\,i({\omega }_{0}+{E}_{0}^{i}){C}_{0}^{i}(t)-\,i\sum _{k}{g}_{k}{C}_{k}(t),\,\frac{d{C}_{l\ne 0}^{i}(t)}{dt}=-\,i({\omega }_{0}+{E}_{l}^{i}){C}_{l\ne 0}^{i},$$

11 $$\frac{d{C}_{k}(t)}{dt}=-\,i{\omega }_{k}{C}_{k}(t)-i\sum _{j=1}^{N}{g}_{k}^{\ast }{C}_{0}^{i}(t).$$

A convenient way to solve the above equations is to use the following redefinitions

12 $$\begin{array}{rcl}{\tilde{C}}_{l}^{i}(t) & = & {e}^{i({\omega }_{0}+{E}_{l}^{i})t}{C}_{l}^{i}(t),\,l=0,1,2,\mathrm{...},M-1,\\ {\tilde{C}}_{k}(t) & = & -\,{e}^{i{\omega }_{k}t}{C}_{k}(t).\end{array}$$

Now, by substituting Eq. (12) into Eqs (10) and (11), we can obtain the following differential equations

13 $$\frac{d{\tilde{C}}_{0}^{i}(t)}{dt}=-\,i\sum _{k}{g}_{k}{e}^{i({\omega }_{0}+{E}_{0}^{i}-{\omega }_{k})t}{\tilde{C}}_{k}(t),\,\frac{d{\tilde{C}}_{l\ne 0}^{i}(t)}{dt}=0,$$

14 $$\frac{d{\tilde{C}}_{k}(t)}{dt}=-\,i{g}_{k}^{\ast }{e}^{-i({\omega }_{0}+{E}_{0}-{\omega }_{k})t}\sum _{i=1}^{N}{\tilde{C}}_{0}^{i}(t).$$

Integrating Eq. (14) and substituting it into Eq. (13) gives the integro-differential equation

15 $$\frac{d{\tilde{C}}_{0}^{i}(t)}{dt}=-\,{\int }_{0}^{t}f(t-t^{\prime} )\sum _{i=1}^{N}{\tilde{C}}_{0}^{i}(t^{\prime} )dt^{\prime} ,$$

where the correlation function *f*(*t* − *t*′) is related to the spectral density *J*(*ω*) of the reservoir by

16 $$f(t-t^{\prime} )={\int }_{0}^{\infty }d\omega J(\omega ){e}^{-i({\omega }_{0}+{E}_{0}^{i}-\omega )(t-t^{\prime} )}.$$

Here, the structure of the common reservoir can be described by an effective Lorentzian spectral density of the form.

17 $$J(\omega )=\frac{1}{2\pi }\frac{{\gamma }_{0}\lambda }{{(\omega -{\omega }_{0})}^{2}+{\lambda }^{2}},$$

where *λ* is the spectral width, *γ*_0_ is the coupling strength, and *ω*_0_ is the central frequency of the reservoir, which is equal to the transition frequency of qubits. Using the Laplace transformation and its inverse, we can obtain a formal solution for ${\tilde{C}}_{0}^{i}(s)$ as

18 $$\begin{array}{rcl}{\tilde{C}}_{0}^{i}(t) & = & {e}^{-(\lambda -i{E}_{0}^{i})t/2}(\cosh (\frac{Dt}{2})+\frac{\lambda -i{E}_{0}^{i}}{D}\,\sinh (\frac{Dt}{2})){\tilde{C}}_{0}^{i}(0)\\  &  & +(1-{e}^{-(\lambda -i{E}_{0}^{i})t/2}(\cosh (\frac{Dt}{2})+\frac{\lambda -i{E}_{0}^{i}}{D}\,\sinh (\frac{Dt}{2})))\frac{{\sum }_{l\ne 0}{\tilde{C}}_{0}^{i}(0)-{\tilde{C}}_{l}^{i}(0)}{N},\end{array}$$

where

19 $$D=\sqrt{{(\lambda -i{E}_{0}^{i})}^{2}-2{\gamma }_{0}\lambda N}.$$

Also we can obtain ${\tilde{C}}_{l\ne 0}^{i}(t)={\tilde{C}}_{l\ne 0}^{i}\mathrm{(0)}$ by considering the second part of Eq. (13). Then, by using Eq. (12), we can acquire the formal solution for the probability amplitudes ${C}_{l}^{i}(t)$ s (*l* = 0, 1, 2, ..., *M* − 1).

Now, we return to the basis {|*i*, *j*〉} and obtain the |*ψ*(*t*)〉 in (9) in terms of this basis as follows

20 $$|\psi (t)\rangle =\xi \mathrm{(0)|}\bar{{\bf{0}}}{\rangle }_{S}\mathrm{|0}{\rangle }_{E}+\sum _{i=1}^{N}\sum _{j=0}^{M-1}{\xi }_{j}^{i}(t)|i,j{\rangle }_{S}\mathrm{|0}{\rangle }_{E}+\sum _{k}{C}_{k}(t)|\bar{{\bf{0}}}{\rangle }_{S}{\mathrm{|1}}_{k}{\rangle }_{E},$$

where

21 $${\xi }_{j}^{i}(t)=\sum _{l=0}^{M-1}\sqrt{\frac{w(l)}{{d}_{l}}}{K}_{l}(j){C}_{l}^{i}(t),$$

is the probability amplitude for the excitation of the *j*th qubit located in the *i*th chain.

In this step, we impose the initial condition in such a way that only the qubit at site 0 of the 1st chain is initially excited, i.e. ${\xi }_{0}^{i\mathrm{=1}}\mathrm{(0)}\ne 0$ and ${\xi }_{j\ne 0}^{i\ne 1}\mathrm{(0)}=0$ with $|\xi {\mathrm{(0)|}}^{2}+|{\xi }_{0}^{i\mathrm{=1}}{\mathrm{(0)|}}^{2}=1$. In fact by the protection process introduced in this paper, we expect that the quantum state $|\psi \rangle =\xi \mathrm{(0)|0}{\rangle }_{S}+{\xi }_{0}^{i\mathrm{=1}}\mathrm{(0)|1}\rangle$ prepared initially at one end of a given chain, for example the 1st one, can be enabled to transfer to the other end of this chain with a high fidelity of state transfer. This is equivalent to the evolution of the state $|\psi (t=0)\rangle =\xi (0)|\bar{0}{\rangle }_{S}+{\xi }_{0}^{i=1}(0)|\mathop{\underbrace{{1}_{1,0}\mathrm{00...0...0}}}\limits_{N\times M}{\rangle }_{S}$ to the target state $|{\psi }_{tar}\rangle =\xi (0)|\bar{0}{\rangle }_{S}+{\xi }_{0}^{i=1}(0)|\mathop{\underbrace{{\mathrm{000...1}}_{1,M-1}\mathrm{...0}}}\limits_{N\times M}{\rangle }_{S}$, at a certain time *t* with a considerable fidelity. On the other hand, to obtain the ${\xi }_{j}^{i\mathrm{=1}}(t)$ in Eq. (21) as an explicit function of time corresponding to the given initial condition, we impose this condition on the ${C}_{l}^{i\mathrm{=1}}(t)$ s at *t* = 0. So, due to the unitarity of $\hat{\bar{U}}$, as denoted in Eq. (4), and using Eq. (21), the following relation is obtained for ${C}_{l}^{i\mathrm{=1}}\mathrm{(0)}$ as

22 $${C}_{l}^{i=1}(0)=\sqrt{\frac{w(0)}{{d}_{l}}}{K}_{l}(0){\xi }_{0}^{i=1}(0).$$

Therefore, the probability amplitude for finding the initial excitation, at time *t*, in the qubit located at site *j* of the 1th chain is given by

23 $${\xi }_{j}^{i=1}(t)={\chi }_{j}^{i=1}(t){\xi }_{0}^{i=1}(0),$$

where

24 $$\begin{array}{rcl}{\chi }_{j}^{i=1}(t) & = & \frac{w(0)}{\sqrt{{d}_{0}{d}_{j}}}{e}^{-i({\omega }_{0}+{E}_{0}^{1})t}\\  &  & \times (\frac{N-1}{N}+\frac{{e}^{-(\lambda -i{E}_{0}^{1})t/2}}{N}(\cosh (\frac{Dt}{2})+\frac{\lambda -i{E}_{0}^{1}}{D}\,\sinh (\frac{Dt}{2})))\\  &  & +\sum _{l=1}^{M-1}\sqrt{\frac{w(0)w(l)}{{d}_{l}{d}_{j}}}{K}_{l}(j){e}^{-i({\omega }_{0}+{E}_{l}^{1})t}.\end{array}$$

After tracing out from the state (20) with respect to the degrees of freedom of the structured reservoir and all of the qubits except the the qubit located at the end of the 1st chain, the reduced density matrix becomes as

25 $${\rho }_{M-1}^{i=1}(t)=(\begin{array}{cc}|{\xi }_{M-1}^{i=1}(t){|}^{2} & {\xi }_{M-1}^{i=1}(t){\xi }^{\ast }(0)\\ {\xi }_{M-1}^{\ast i=1}(t)\xi (0) & 1-|{\xi }_{M-1}^{i=1}(t){|}^{2}\end{array}).$$

Consequently, the fidelity between the state (25) and the state |*ψ*〉 is obtained as

26 $$\begin{array}{rcl}F(|\psi \rangle \langle \psi |,{\rho }_{M-1}^{i=1}(t)) & = & \sqrt{\langle \psi |{\rho }_{M-1}^{i=1}(t)|\psi \rangle }\\  & = & \sqrt{|\xi (0){|}^{2}(1-2|{\xi }_{M-1}^{i=1}(t){|}^{2}+{\xi }_{M-1}^{i=1}(t){\xi }_{0}^{\ast i=1}(0)+{\xi }_{M-1}^{\ast i=1}(t){\xi }_{0}^{i=1}(0))+|{\xi }_{M-1}^{i=1}(t){|}^{2}}.\end{array}$$

Since the $|\bar{{\bf{0}}}{\rangle }_{S}$ component of the the state |*ψ*(0)〉 is invariant under the evolution, it suffices to focus to the choice *ξ*(0) = 0 and ${\xi }_{0}^{i\mathrm{=1}}\mathrm{(0)}=1$. Therefore, it is concluded that the fidelity of state transfer for an excitation between two ends of the dissipative spin chain in the presence of other *N* − 1 similar auxiliary chains contained in the reservoir is written as

27 $$|\,{f}_{0,M-1}(t)|=|{\xi }_{M-1}^{i=1}(t)|=|{\chi }_{M-1}^{i=1}(t)|.$$

Now by employing the Eq. (27) and following ref.^1^, we can calculate the average fidelity over all pure input states as

28 $$F(t)=\frac{1}{2}+\frac{|\,{f}_{0,M-1}(t){|}^{2}}{6}+\frac{|\,{f}_{0,M-1}(t)|\,\cos (\gamma )}{3},$$

where *γ* = *Arg*(*f*_0,*M*−1_(*t*)) is the argument of the complex quantity *f*_0,*M*−1_(*t*). Figures (2), (3) and (4) demonstrate the performance of the QST protocol introduced in this paper for the chains with length of *M* = 2, 3, 4. Figure 2(a) and (b) shows the QST efficiency represented in terms of the fidelity and maximum average fidelity of state transfer for a two-qubit spin chain, i.e. *M* = 2 respectively. For this case, in the absence of additional chains, i.e. *N* = 1, the fidelity of state transfer in Eq. (27) is strongly affected by the dissipation. A steady value is observed for the fidelity of state transfer. In fact, the interaction of the two-qubit spin chain with the common reservoir is established only through the respective eigenstate $|{{\rm{\Phi }}}_{0}\rangle =\mathrm{1/}\sqrt{2}\mathrm{(|10}\rangle +\mathrm{|01}\rangle )$, and the other eigenstate $|{{\rm{\Phi }}}_{1}\rangle \mathrm{=1/}\sqrt{2}\mathrm{(|10}\rangle -\mathrm{|01}\rangle )$ is decoupled from the reservoir. Since $|\psi (0){\rangle }_{S}=|10{\rangle }_{S}=\mathrm{1/}\sqrt{2}(|{{\rm{\Phi }}}_{0}\rangle +|{{\rm{\Phi }}}_{1}\rangle )$, therefore $|\,{f}_{0,1}(t\to \infty )|=\frac{1}{2}$, as shown in Fig. 2. On the other hand, in the presence of additional chains (for example *N* = 50), a considerable improvement is observed in the efficiency of state transfer (see Fig. 2(a) and (b)). Evidently, whatever *N* becomes larger, the state transfer process in the mentioned chain is better protected against the dissipative noises.

Figure 3(a) and (b) shows the fidelity and maximum average fidelity of the quantum state transfer along a spin chain with three qubits. In the absence of the additional chains, the initial state is $$|\psi (0){\rangle }_{S}=|100{\rangle }_{S}=$$$$\frac{1}{2}|{{\rm{\Phi }}}_{0}\rangle +\frac{1}{2}|{{\rm{\Phi }}}_{1}\rangle +\frac{1}{\sqrt{2}}|{{\rm{\Phi }}}_{2}\rangle $$, where $$|{{\rm{\Phi }}}_{0}\rangle =\frac{1}{2}(|100\rangle +\sqrt{2}|010\rangle +|001\rangle )$$, $$|{{\rm{\Phi }}}_{1}\rangle =\frac{1}{2}(|100\rangle -\sqrt{2}|010\rangle +|001\rangle )$$ and $$|{{\rm{\Phi }}}_{2}\rangle =\frac{1}{\sqrt{2}}(|100\rangle -|001\rangle )$$ are the eigenstates of the three-qubit chain. Obviously, |*ψ*(0)〉_*s*_ has a support on the decoupled subspace spanned by $${\{|{{\rm{\Phi }}}_{1}\rangle }_{S},|{{\rm{\Phi }}}_{2}{\rangle }_{S}\}$$, so the interaction of the three-qubit spin chain with the reservoir is possible only through the eigenstate |Φ_0_〉. Therefore, by entering the corresponding three-qubit auxiliary chains (*N* = 45), the QST for the three-qubit spin chain can be well-protected against the noises. This procedure can be repeated for the four-qubit chain by considering the four-qubit auxiliary chains (*N* = 40), as depicted in Fig. 4(a) and (b).

To explain why the presence of auxiliary chains in the reservoir leads to the protection of QST in the considered chain, let us remember from refs^36–38^ that the protection of entanglement or coherence in a qubit (2-dimensional) system or in a qutrit (3-dimensional) system, is achieved by entering auxiliary qubit or qutrits, into the related reservoir respectively. In fact, entering auxiliary systems into the respective reservoirs leads to more separation of system-reservoir bound state, as an isolated eigenstate of the whole system, from the remainder spectrum. This approach can be extended for protection of an open quantum system with M-dimensional Hilbert space, which could be considered as a spin chain with *M* spin in the single excitation subspace. Therefore, entering the other similar auxiliary chains into the reservoir leads to improvement of the formed bound state, i.e, a better separation of it from the remainder spectrum. Consequently, this situation gives the protection process for the mentioned spin chain with length of *M*.

It should be noted that a common reservoir with Lorentzian spectral density makes it possible to solve the dynamics of the open system (system with *N* identical chains) analytically. While, if we take for example Ohmic spectral density, it is not possible to obtain an exact master equation for the dynamics of the considered system. Also, it is impossible to obtain an exact dynamics for the system by taking local environments interacting individually with the chains. In addition, we note that the protection process introduced in this paper has similar performance for Markovian and Non-Markovian noises (see, for example^36^).

## Conclusion

In summery, we investigated a mechanism for the protection of the intrinsic PST of a pre-engineered linear spin chain in the presence of dissipative noises. By obtaining the exact dynamics, it was shown that the protection process can be well-controlled through the entering non-interacting auxiliary chains into the structured reservoir and therefore, high fidelity state transmission is achievable in the considered spin chain. Furthermore, it was illustrated that the protocol has better efficiency for the chains with more qubits.

## Methods

### Perfect State transfer for an isolated spin chain

We consider a set of *M* identical qubits on a linear chain with nearest-neighbor *XY* coupling. The Hamiltonian of the system is given by

29 $$\hat{H}={\omega }_{0}\sum _{j=0}^{M-1}{\sigma }_{j}^{+}{\sigma }_{j}^{-}+\sum _{j=0}^{M-2}{J}_{j}({\sigma }_{j}^{+}{\sigma }_{j+1}^{-}+{\sigma }_{j}^{-}{\sigma }_{j+1}^{+})$$

where *ω*_0_ is the transition frequency and *J*_*j*_ is the coupling strength between the qubits located at site *j* and *j* + 1. The lowering operator ${\sigma }_{j}^{-}={({\sigma }_{j}^{+})}^{\dagger }=|0\rangle \langle j|$ describes decay from the excited state $|j\rangle \equiv |\mathop{\underbrace{{\mathrm{000...1}}_{j}\mathrm{...0}}}\limits_{M}\rangle$ into the ground state $|{\bf{0}}\rangle \equiv |\mathop{\underbrace{\mathrm{000...0...0}}}\limits_{M}\rangle$, where |*j*〉 describes the state in which, there is an excitation in the qubit located at the site *j* (*j* = 0, 1, 2, ..., *M* − 1). The states {|*j*〉} are considered as a set of basis for the single excitation subspace of the spin chain, called as canonical or standard basis.

The matrix representation of the Hamiltonian (28) in this basis takes the following form

30 $$\hat{H}=(\begin{array}{cccccc}{\omega }_{0} & {J}_{0} & 0 & \mathrm{...} & 0 & 0\\ {J}_{0} & {\omega }_{0} & {J}_{1} & \mathrm{...} & 0 & 0\\ 0 & {J}_{1} & {\omega }_{0} & \mathrm{...} & 0 & 0\\ . & . & . & . & . & .\\ . & . & . & . & . & .\\ . & . & . & . & . & .\\ 0 & 0 & 0 & \mathrm{...} & {\omega }_{0} & {J}_{M-1}\\ 0 & 0 & 0 & \mathrm{...} & {J}_{M-1} & {\omega }_{0}\end{array}).$$

This Hamiltonian is real and symmetric, so from the spectral theorem^39^ it can be written as

31 $$\hat{H}=\hat{U}\hat{D}{\hat{U}}^{T}$$

where $\hat{D}$ is a diagonal matrix and $\hat{U}$ is an orthogonal one as

32 $$\hat{D}={\rm{diag}}({E}_{0},{E}_{1},\mathrm{...},{E}_{M-1}),\,\,\hat{U}{\hat{U}}^{T}={\hat{U}}^{T}\hat{U}=1,$$

by noting that *E*_*j*_ s denote energy eigenvalues of $\hat{H}$, and *T* as the transpose operation. The columns of the matrix $\hat{U}$ are the eigenvectors of $\hat{H}$ as

33 $$|{\varphi }_{l}\rangle =(\begin{array}{c}{U}_{0l}\\ {U}_{1l}\\ .\\ .\\ .\\ {U}_{(M-1)l}\end{array})=\sum _{j=0}^{M-1}{U}_{jl}|j\rangle ,\,(l=0,1,\mathrm{...},M-1)$$

with $\hat{H}|{\varphi }_{l}\rangle ={E}_{l}|{\varphi }_{l}\rangle$. From the orthogonality of $\hat{U}$, the inverse relation reads

34 $$|j\rangle =\sum _{l=0}^{M-1}{U}_{jl}|{\varphi }_{l}\rangle .$$

The dynamics of the system is described by the unitary time evolution operator $\hat{{\mathscr{U}}}(t)\equiv exp(-\,i\hat{H}t)$. We assume that there is an excitation at site 0 (*j* = 0) of the chain at *t* = 0, which we desire to transfer it to site *M* − 1. After a certain time *t* the system evolves to the state $\hat{{\mathscr{U}}}(t\mathrm{)|0}\rangle \,({\rm{or}}\,\hat{{\mathscr{U}}}(t)|j=0\rangle =\hat{{\mathscr{U}}}(t)|\mathop{\underbrace{\mathrm{100...0...0}}}\limits_{M}\rangle )$ which, in general, is a superposition of various standard states |*j*〉 s. So, the transition amplitude for an excitation in transferring from one end of the chain to the other end is given by the transfer fidelity or *fidelity of state transfer* as follows

35 $${f}_{0,M-1}(t)=\langle M-1|\hat{{\mathscr{U}}}(t)|0\rangle .$$

Substituting Eqs (32) and (33) into Eq. (34) gives

36 $${f}_{0,M-1}(t)=\sum _{l=0}^{M-1}{U}_{0,l}{U}_{M-1,l}{e}^{-i{E}_{l}t}.$$

The situation of PST at time *t* from one end of the chain to the other one occurs when | *f*_0,*M*−1_(*t*)| = 1. This can be accomplished through the proper choice of coupling strength *J*_*j*_ between the adjacent qubits. If the coupling strengths of the spin chain are chosen to be uniform, the PST does not occur for chains containing more than three qubits^6,9^. However, it is known that by choosing the coupling strengths as ${J}_{j}=\sqrt{(j+1)(M-j-1)}$ (*j* = 0, 1, ..., *M* − 1), the PST is achievable^6,31,40^. It turns out that in this sense, the eigenvectors of $\hat{H}$ (the columns of $\hat{U}$) are related to the well-known *Krawtchouk polynomial*^9,31^ as

37 $$|{\varphi }_{l}\rangle =\sum _{j=0}^{M-1}{U}_{jl}|j\rangle =\sum _{j=0}^{M-1}{\tilde{K}}_{l}(j)|j\rangle ,$$

where ${\tilde{K}}_{l}(j)$ is the orthonormal Krawtchouk function defined as

38 $${\tilde{K}}_{l}(j)\equiv \frac{\sqrt{w(j)}{K}_{l}(j)}{\sqrt{{d}_{l}}},$$

where *K*_*l*_(*j*) is the Krawtchouk polynomial^41–43^ of degree *l* (*l* = 0, 1, ..., *M* −1) in the variable *j*, with parameter 0 < *p* < 1, written as follows

39 $${K}_{l}(j)={F}_{1}(-j,-\,l;-\,M+1;\frac{1}{p}).$$

The function *F*_1_ is the classical hypergeometric series^44^ and in this case, it is a terminating series because of the appearance of the negative −*l* as a numerator parameter. The *w*(*j*) is the weight function in *j*, and *d*_*l*_ is a function depending on *l* as

40 $$w(j)=(\begin{array}{c}M-1\\ j\end{array}){p}^{j}{(1-p)}^{M-1-j},\,{d}_{l}=\frac{1}{(\begin{array}{c}M-1\\ l\end{array})}{(\frac{1-p}{p})}^{l},$$

where according to the aim of this paper, we set $p=\frac{1}{2}$. Also the corresponding energy eigenvalues of $\hat{H}$ are

41 $${E}_{l}=M-\,1-\,2l.$$

Let us consider that there is an excitation at first qubit of the chain, while the others are in the ground state. By substituting the Eqs (37) and (41) into Eq. (36), one can compute the fidelity of state transfer for an excitation from site 0 to *M* − 1 as

42 $$|\,{f}_{0,M-1}(t)|=|\,\sin (t){|}^{M-1}.$$

Eq. (41) gives the perfect state transfer between two ends of the chain with transfer time $t=\frac{\pi }{2}$.
